# Annotation and characterization of immunoglobulin loci and CDR3 polymorphism in water buffalo (*Bubalus bubalis*)

**DOI:** 10.3389/fimmu.2024.1503788

**Published:** 2025-01-20

**Authors:** Yunlan Deng, Fengli Wu, Qianqian Li, Lidie Yao, Chengzhi Yang, Long Ma, Xinsheng Yao, Jun Li

**Affiliations:** ^1^ Department of Immunology, Center of Immunomolecular Engineering, Innovation & Practice Base for Graduate Students Education, Zunyi Medical University, Zunyi, China; ^2^ Department of Laboratory, The Affiliated Yongchuan Hospital of Chongqing Medical University, Chongqing, China

**Keywords:** buffalo, immunoglobulin, CDR3 repertoire, ultra-long CDR3 sequences, V(D)J recombination

## Abstract

**Introduction:**

Immunoglobulin (Ig) refers to the nomenclature for all antibody proteins produced by B lymphocytes. The genetic locus encoding Ig is critical for vertebrate humoral immune responses and diverse antibody repertoires. Despite the critical role of buffaloes as livestock and their significance in disease transmission, the Ig loci of this species have not been thoroughly annotated. This study aimed to systematically characterize the Ig loci in buffaloes and their unique features, providing a foundation for understanding buffalo immune function.

**Methods:**

The genomic assembly of Murrah buffalo (NDDB_SH_1) was analyzed to annotate Ig loci. Annotation criteria included functional motifs, RSS sequences, and structural features of V, D, J, and C genes. The CDR3 repertoires were constructed using genomic DNA extracted from spleen samples of five healthy buffaloes. High-throughput sequencing of multiplex PCR products enabled repertoire analysis, and MiXCR software was used for alignment and CDR3 extraction. Repertoire diversity, gene usage, and clonal frequencies were analyzed using the Immunarch R package.

**Results:**

The IgH locus spans approximately 667 kb on chromosome 20, containing two D-J-C clusters, 54 VH genes, 10 DH genes, 8 JH genes, and 9 CH genes. The Igκ locus, located on chromosome 12, encompasses 24 Vκ genes, 5 Jκ genes, and 1 Cκ gene, while the Igλ locus on chromosome 17 includes 71 Vλ genes, 3 Jλ genes, and 3 Cλ genes. We also conducted a detailed examination of the buffalo IgH CDR3 repertoire, revealing the presence of ultra-long CDR3 sequences, a biased usage of certain V genes, and a high-frequency usage of IgHJ1-4 genes. Furthermore, we identified a set of shared clonotypes across the samples, highlighting commonalities in the buffalo antibody repertoire.

**Conclusion:**

These findings contribute to the understanding of buffalo immune function and provide insights into the evolution and diversity of ruminant immunoglobulin genes.

## Introduction

Buffalo (*Bubalus bubalis*) is a major component of the livestock industry worldwide, serving not only as draught animals but also as a significant source of milk and meat products (1, 2). Despite their economic importance, there has been limited progress in understanding the mechanisms that regulate their immune responses to pathogens and parasites, which critically affect their health and productivity. Studies have shown that buffalo can mount robust humoral immune responses against a wide range of viruses, bacteria, and parasites, as demonstrated by various serological studies (3–6). However, the genetic and molecular basis underpinning the development and evolution of their immunoglobulin (Ig) repertoires and antibody responses remains largely uncharacterized.

Ig is composed of heavy and light chains, with the heavy chain formed through VDJ recombination at the heavy chain locus, while the light chain is generated through VJ recombination at either the kappa (κ) or lambda (λ) light chain loci (7). The specific recognition and binding of pathogens by antibodies rely both on the unique combinations of heavy and light chains and the inherent diversity created by the VDJ and VJ recombination processes (8). This allows B cells to generate a vast array of antigen-binding sites, enabling them to specifically identify and target a wide range of pathogens (9). The genomic organization and diversity of Ig in herbivores have been characterized in cattle (10, 11), sheep (12), yak (13), horse (14) and goat (15), and the germline genes encoding the heavy and light chains of Ig in these ruminants, exhibit notable differences. The differences of Ig gene organization in these ruminants suggest potential variations in their immune evolution and defense mechanisms (16). This highlights the importance of investigating each species individually to better understand how these genetic differences may influence their unique immune responses and adaptations to pathogens.

The complementarity-determining region (CDR) regions of the immunoglobulin heavy and light chains are key sites for antibody-antigen binding, particularly the CDR3 region, which exhibits the greatest structural and sequence diversity (17). Deciphering the germline gene organization and CDR3 repertoire characteristics of Ig in buffalo not only contributes to understanding their immune defense mechanisms but also provides valuable insights for vaccine development and disease-resistant breeding (18, 19). In this study, we annotated and presented the comprehensive analysis of germline gens the Ig heavy (IgH), kappa (Igκ), and lambda (Igλ) chains in buffalo, along with a detailed characterization of the CDR3 regions of the IgH chain in the spleen of five buffaloes. By annotating the germline gene segments and analyzing the CDR3 repertoire, we provide a foundation for exploring the adaptive immune responses in buffalo, which could inform future efforts in enhancing disease resistance and improving vaccine design tailored to this species.

## Materials and methods

### Annotation of buffalo immunoglobulin genes

The genomic assembly sequence of the Murrah breed of buffalo (NDDB_SH_1, NCBI Accession No: GCF_019923935.1, 25 chromosome pairs, total genome size 2.6 Gb) was obtained from the NCBI website (https://www.ncbi.nlm.nih.gov/). To identify the specific chromosomal locations of the buffalo Ig germline genes, we downloaded the existing germline C gene sequences and V gene sequences (in FASTA format) for all species from the IMGT database (https://www.imgt.org/genedb/). All germline C genes and V genes were subjected to BLAST analysis (https://blast.ncbi.nlm.nih.gov/Blast.cgi) against the buffalo chromosome assembly sequences to determine the chromosomal positions of the buffalo Ig locus.

Using Geneious Prime (Version: 2023.2.1), we aligned the germline C gene sequences downloaded from the IMGT database with the buffalo Ig locus sequences obtained from the NCBI database through the "Map to Reference" function. Sequences with high nucleotide similarity were mapped onto the chromosome sequences. Based on the alignment results, potential germline genes were identified. This approach allowed for the annotation of the buffalo V, D, J, and C genes. To validate the mapped sequences and to identify any unannotated V, D, J, and C genes, we referenced the 12/23 recombination signal sequences (RSS) of humans and mice. All RSS sequences within the buffalo Ig locus sequences were retrieved from the RSS Database (https://www.itb.cnr.it/rss/analyze.html) and annotated to the corresponding suspected germline gene sequences. Subsequently, the buffalo Ig locus sequences were input into IMGT/LIGMotif (https://www.imgt.org/ligmotif) to further investigate unannotated germline genes based on the sequences filtered by mapping and RSS identification. The alignment results from the IMGT/LIGMotif tool were utilized for analysis and validation of the annotated sequences.

Using the Ig germline genes from cattle in the IMGT database as a reference, we annotated the buffalo Ig loci by identifying the specific characteristics of the V, D, J, and C genes. The following criteria were employed for filtering the annotated V genes: (1) presence of a complete translation initiation codon; (2) existence of appropriate donor/acceptor splice sites; (3) sequence length approximately around 290 bp; (4) corresponding RSS sequence at the 3' end. D genes were primarily characterized by G-rich nucleotide sequences. J gene nucleotide sequences were approximately 50 bp in length and contained conserved WGXG amino acid motifs. C genes, the IgH locus contains 9 CH genes, each comprising 1 to 4 exons, while the Igκ and Igλ loci each contain a single C gene with a single exon.

### Functional analysis and classification of buffalo Ig genes

The functional discrimination and feature analysis of the buffalo Ig germline genes were performed according to the IMGT annotation guidelines and rules (20, 21). The annotated V genes were uploaded to IMGT/V-QUEST (https://www.imgt.org/IMGT_vquest/input) for analysis. Based on the compositional features of the V genes and the functional classification rules established by IMGT, the V genes were categorized into three classes: (1) functional genes (F), (2) open reading frames (ORF), and (3) pseudogenes (P). Using the Clustal Omega function within Geneious Prime, we conducted nucleotide and amino acid similarity analyses of the annotated germline V genes, marking the framework region (FR), CDR, and conserved amino acid residues. The V genes were named based on their homology to cattle V genes, following the IMGT rules for family classification with a nucleotide similarity threshold of 75%. To reflect the physical arrangement within the buffalo locus, we named the V genes sequentially from 5' to 3'. D, J, and C genes were similarly named according to their respective positions in the locus from 5' to 3'. The Weblogo3 tool (https://weblogo.threeplusone.com/create.cgi) was utilized to analyze the RSS sequences of the buffalo V and J genes, generating logo diagrams to compare the compositional features and conservation of the buffalo RSS sequences against those in the IMGT database.

### Sample collection and IgH CDR3 repertoire construction

Spleen tissue samples from five healthy adult buffaloes (breed:Murrah) were collected in June 2022 from a slaughterhouse in Nanning, Guangxi Province. All samples passed veterinary quarantine and were immediately frozen in liquid nitrogen for preservation. All experimental procedures were conducted in accordance with the Regulations on the Administration of Affairs Concerning Experimental Animals approved by the State Council of the People’s Republic of China. This study was approved by the Institutional Animal Care and Use Committee of Zunyi Medical University.

The IgH CDR3 repertoire was constructed using a multiplex PCR approach. Genomic DNA was extracted from the buffalo spleen samples using the DNeasy Blood & Tissue Kit (QIAGEN GmbH, Germany). Primers (**Supplementary Tables 1**, **2**) were synthesized by Sangon Biotech (Shanghai, China), and PCR was performed with DreamTaq Green PCR Master Mix (2X) (Thermo Fisher Scientific, Baltics). The reaction mix was prepared with the primers and PCR conditions were set as follows: initial denaturation at 95°C for 1 minute, denaturation at 95°C for 30 seconds, annealing at 58.5°C for 30 seconds (40 cycles), extension at 72°C for 40 seconds, and a final extension at 72°C for 10 minutes. PCR products were visualized using agarose gel electrophoresis, where a clear band between 200-300 bp was excised (**Supplementary Figures 1**, **2**). Gel-purified PCR products were subsequently sequenced using high-throughput sequencing technology (BGI, China).

### Construction of the buffalo IgH germline reference library

Following the methodology outlined in our previous publications (22, 23), we briefly summarize the construction of the germline reference library as follows: Based on the annotation of the IgH gene locus, we compiled a total of 54 IgHV genes, 10 IgHD genes, 8 IgHJ genes, and 9 IgHC genes. Using Geneious software, the amino acid sequences of the V and J genes were aligned and structurally segmented. Based on the conserved amino acid motifs in the FRs and CDRs recognized by MiXCR, in alignment with IMGT database definitions, the sequences were segmented into four FRs (FR1, FR2, and FR3 within the V gene backbone, and FR4 within the J gene backbone) and three CDRs (CDR1, CDR2, and CDR3). These FRs represent the structural framework of the immunoglobulin variable regions at the protein level, as determined through amino acid sequence alignment. This segmented information, combined with the nucleotide sequences of the germline genes, was used to generate a reference library that MiXCR can recognize and utilize. This reference library was employed to align the buffalo IgH high-throughput sequencing (HTS) data, enabling the extraction of CDR3 sequences for further analysis.

### The analysis of buffalo IgH CDR3 repertoire

Following the construction of the IgH CDR3 library and sequencing, the obtained sequencing data were analyzed using the MiXCR software, in conjunction with our custom-built buffalo Ig reference library. The MiXCR software was utilized to align the sequencing reads to the reference database, enabling the extraction of CDR3 sequences, gene usage, and other relevant features from the buffalo IgH repertoire (24). Once the initial sequence alignment and annotation were completed, the data for each sample were manually inspected to remove any sequences that contained stop codons within the CDR3 region. This step ensured the accuracy of the functional CDR3 sequences being analyzed. The processed data were analyzed using the Immunarch package in the R programming language. Clonality analysis were performed based on the total clone counts for each sample, assessing the overall repertoire diversity and the distribution of clonal frequencies. Simultaneously, CDR3 length distribution and germline V, D, and J gene usage were examined using unique clones.

## Results

### Structure of the genomic organization of buffalo IgH

**Figure 1:**
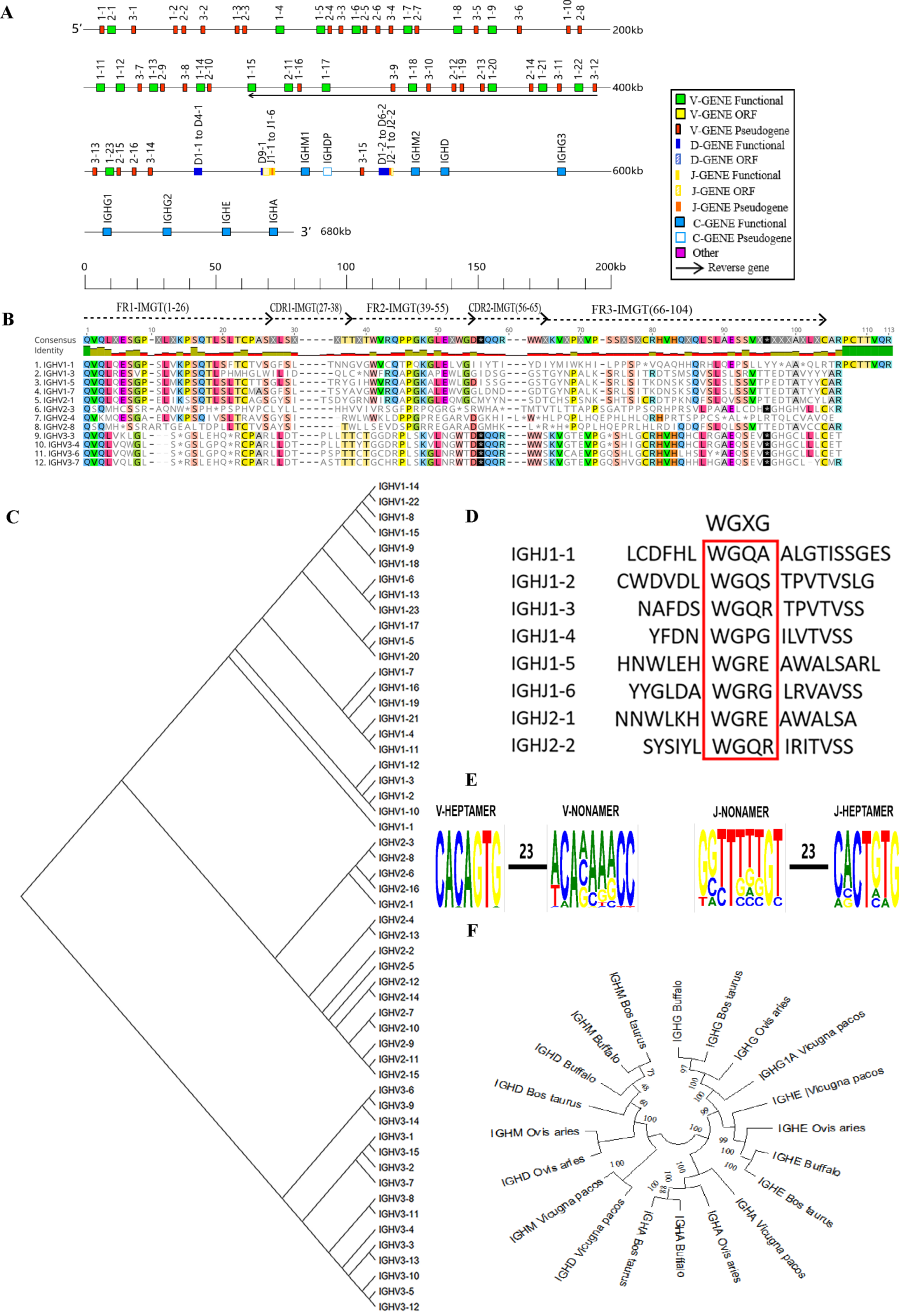
Schematic structure of the genomic organization of buffalo IgH and its relatedness with representative vertebrate species. **(A)** Schematic structure of the genomic organization of buffalo IgH; **(B)** The sequence diversity of buffalo VH genes; **(C)** The phylogenetic tree of buffalo VH; **(D)** The sequence diversity of buffalo JH genes; **(E)** Conservation and mutation of 23RSS and 23RSS sequences in buffalo VH and JH genes; **(F)** The phylogenetic tree of different CH gene clusters between buffalo and representative vertebrate species(cattle, sheep, and alpaca).Built using the N-J method in MEGA11, 1000 bootstrap tests.

The buffalo IgH locus was mapped to chromosome 20 (GenBank: CM034290.1 NC_059176.1) with a total length of approximately 667 kb. This locus contains two D-J-C clusters, including 54 VH genes, of which 35 are pseudogenes (P) and 19 are functional genes (F), along with 10 functional DH genes, 8 JH genes (2 P, 2 F, and 4 ORF), and 9 CH genes (1 P and 8 F) (**Table 1**, **Figure 1A**, **Supplementary Table 3**, **Supplementary Figure 3A**). Using the Geneious Prime software's Clustal Omega tool, we aligned the amino acid sequences of the buffalo IgHV genes according to the structural characteristics of mammalian Ig in the IMGT database. We found that nearly all functional V genes displayed the classical conserved amino acid residues within the framework regions FR1, FR2, and FR3: a cysteine (C) at position 23, a tryptophan (W) at position 41, and a cysteine (C) at position 104. In pseudogenes, mutations in the nucleotide sequences of these conserved positions led to changes in the corresponding amino acids. Additionally, most V genes featured a conserved leucine (L) at position 89 (**Figure 1B**, **Supplementary Figure 4A**).

**Table 1:** Summary of the annotated immunoglobulin gene segments in buffalo.

	IgH				Igκ			Igλ		
	IgHV	IgHD	IgHJ	IgHC	IgκV	IgκJ	IgκC	IgλV	IgλJ	IgλC
F	19	10	2	8	12	1	1	31	2	2
P	35	–	2	1	12	4	–	35	–	1
ORF	–	–	4	–	–	–	–	5	1	–
Total	54	10	8	9	24	5	1	71	3	3

The table includes counts of functional (F), pseudogenes (P), and open reading frames (ORF) for IgH, Igκ, and Igλ chains, along with their respective variable (V), diversity (D), joining (J), and constant (C) genes.

Buffalo VH genes were categorized into three families: family 1, family 2, and family 3 (**Figure 1C**). Most of the functional VH genes were clustered in family 1. In family 3, VH genes did not exhibited the conserved tryptophan (W) at position 41 and contained stop codons, rendering them pseudogenes (**Figure 1B**, **Supplementary Figure 4A**). Based on the IMGT functional classification of bovine J genes, we identified four JH genes (IgHJ1-1, IgHJ1-2, IgHJ1-3, IgHJ2-2) as ORF, lacking the conserved "WGXG" motif. Two JH genes (IgHJ1-5, IgHJ2-1) were identified as pseudogenes due to the absence of donor-splice sites, while IgHJ1-4 and IgHJ1-6 were classified as functional (**Figure 1D**).

RSSs are an essential part of V(D)J recombination. each RSS signal can be dissected into three components: a conserved heptamer (consensus: 5’-CACAGTG) and a conserved nonamer (consensus: 5’-ACAAAAACC), separated by a poorly conserved spacer of either 12 ± 1 or 23 ± 1 bp (25). Analysis of the 23RSS sequences in the V genes and the 23RSS sequences in the J genes revealed that the 7-mer in the buffalo IgHV genes' 23RSS was relatively conserved, but the A residues at positions 4, 6, and 7 of the 9-mer showed lower conservation with multiple mutations. In the buffalo IgHJ genes' 23RSS, the 7-mer was relatively conserved, while the nucleotide at position 3 of the 9-mer was less conserved (**Figure 1E**). A comparative homology analysis of the CH1 region of buffalo IgHC genes with those of other ruminants, including cattle, sheep, and alpaca, was conducted, as CH1 represents a conserved and functionally significant region within the IgHC genes, making it ideal for cross-species comparison. Phylogenetic analysis (**Figure 1F**) revealed that buffalo IgHC genes exhibit the highest similarity to cattle, with a secondary close relationship to sheep.

### Structure of the genomic organization of buffalo Igκ

**Figure 2:**
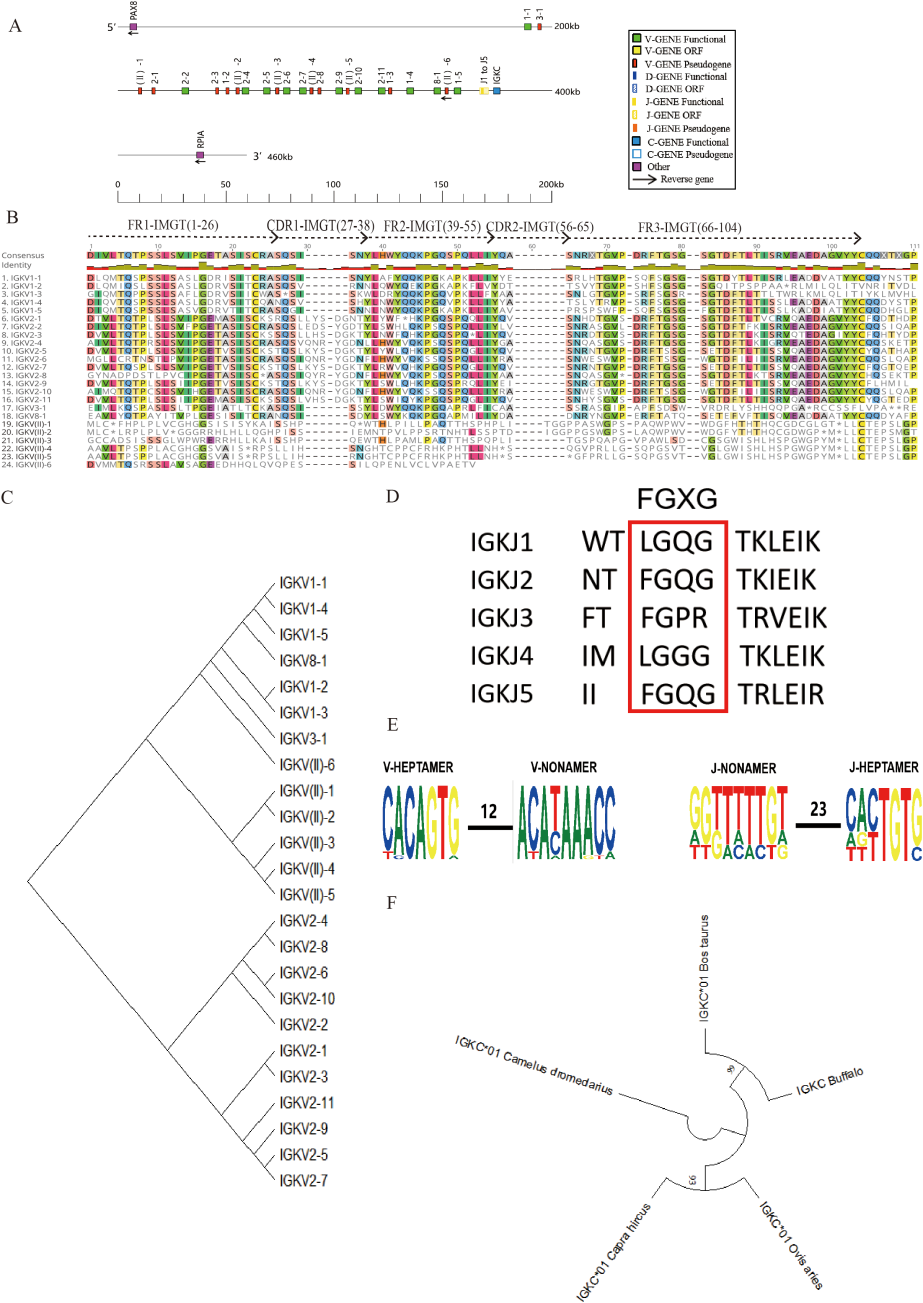
Schematic structure of the genomic organization of buffalo Igκ and its relatedness with representative vertebrate species. **(A)** Schematic structure of the genomic organization of buffalo Igκ; **(B)** The sequence diversity of buffalo Vκ genes; **(C)** The phylogenetic tree of buffalo Vκ; **(D)** The sequence diversity of buffalo Jκ genes; **(E)** Conservation and mutation of 12RSS and 23RSS sequences in buffalo Vκ and Jκ genes; **(F)** The phylogenetic tree of different Cκ gene clusters between buffalo and representative vertebrate species(cattle, sheep,goat and alpaca).Built using the N-J method in MEGA11, 1000 bootstrap tests.

The buffalo Igκ locus is located on chromosome 12 (GenBank: CM034282.1, NC_059168.1), with the identified Ig germline genes spanning a total length of 182 kb. The full locus, including intergenic regions and boundary genes, spans approximately 460 kb (**Figure 2A**, **Supplementary Figure 3B**). This locus contains 24 Vκ genes, of which 12 are potentially functional, while the other 12 are classified as pseudogenes due to the presence of stop codons or the absence of conserved residues (**Figure 2B**, **Table 1**, **Supplementary Table 3**). Comparative analysis of buffalo Vκ genes with IMGT bovine Vκ genes revealed five families: family 1, family 2, family 3, family 8, and clan II (**Figure 2C**). Notably, all Vκ genes in clan II are pseudogenes. Following the Vκ genes, there are five Jκ genes, of which only one, IgκJ2, is functional. The remaining four Jκ genes lack the classical J-heptamer or do not contain the conserved “FGXG” motif, leading to their classification as ORFs (**Figure 2D**).

Analysis of the 12RSS sequences in the V genes and the 23RSS sequences in the J genes of the buffalo Igκ locus revealed that the 7-mer of the 12RSS in the buffalo IgκV genes is relatively conserved, while the conservation of the A residue at position 4 of the 9-mer is low, with mutations to T or C observed. Similarly, the 7-mer of 23 RSS showed good conservation, with more poly A mutations and poor conservation at positions 3 – 7 of the 9-mer. (**Figure 2E**). Homology analysis of the buffalo IgκC gene compared to those of other herbivores, including cattle, sheep, camel, and goat, demonstrated that the buffalo IgκC gene shares the highest homology with cattle, reaching 100%, followed by sheep and goat (**Figure 2F**).

### Structure of the genomic organization of buffalo Igλ

**Figure 3:**
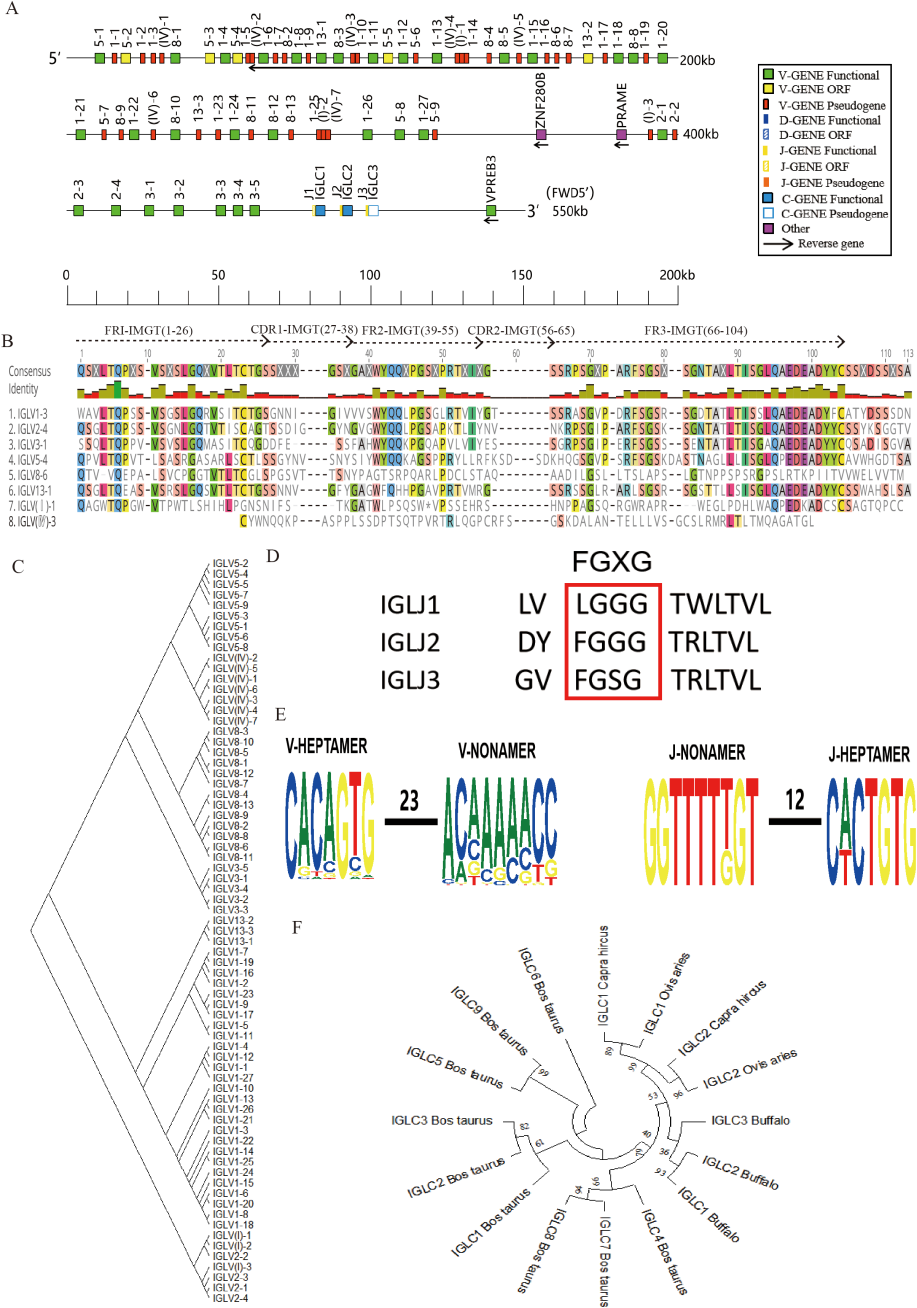
Schematic structure of the genomic organization of buffalo Igλ and its relatedness with representative vertebrate species. **(A)** Schematic structure of the genomic organization of buffalo Igλ; **(B)** The sequence diversity of buffalo Vλ genes; **(C)** The phylogenetic tree of buffalo Vλ; **(D)** The sequence diversity of buffalo Jλ genes; **(E) **Conservation and mutation of 23RSS and 12RSS sequences in buffalo Vλ and Jλ genes; **(F)** The phylogenetic tree of different Cλ gene clusters between buffalo and representative vertebrate species.(cattle, sheep, and goat).Built using the N-J method in MEGA11, 1000 bootstrap tests.

The buffalo Igλ gene is located on chromosome 17 (GenBank: CM034287.1, NC_059173.1), encompassing three J-C clusters and spanning a total length of 474 kb (**Figure 3A**, **Supplementary Figure 3C**). Within the buffalo Igλ locus, a total of 71 Vλ genes were identified, comprising 35 pseudogenes, 31 functional genes, and 5 ORF (**Table 1**, **Supplementary Table 3**). Interestingly, we observed that the transcriptional orientation of 24 Vλ genes is reversed, proceeding in the opposite direction to the typical 5' to 3' orientation. Additionally, three Jλ genes were found, including one ORF and two functional genes, along with three C genes (two functional and one pseudogene). We performed an alignment analysis of the amino acid sequence of the buffalo IgλV genes, and all the functional V genes showed classical conserved amino acid residues: C at 23, W at 41, and C at 104 position. In pseudogenes, the corresponding conserved sites are lacking(**Figure 3B**, **Supplementary Figure 4B**). The buffalo Vλ genes were classified into eight families: family 1, family 2, family 3, family 5, family 8, family 13, family I, and family IV, with all Vλ genes in families I and IV designated as pseudogenes (**Figure 3C**, **Supplementary Figure 4B**). The J genes are located upstream of each C gene, with a total of three J genes identified. IgλJ1 was classified as an ORF due to the absence of the classical J-heptamer and the conserved “FGXG” motif, while IgλJ2 and IgλJ3 were confirmed as functional J genes (**Figure 3D**).

Analysis of the 23RSS sequences in the V genes and the 12RSS sequences in the J genes of the buffalo Igλ locus revealed that the 7-mer of the 23RSS in buffalo IgλV genes is relatively conserved, while the conservation of the A residues at positions 3-7 of the 9-mer is lower, with multiple mutations observed. In contrast, both the 7-mer and 9-mer of the 12RSS in the IgλJ genes showed high conservation (**Figure 3E**). Furthermore, an analysis of the buffalo IgλC genes indicated that IgλC1 and IgλC2 exhibit higher homology, with buffalo displaying greater similarity to sheep and goat compared to other ruminants such as cattle (**Figure 3F**).

### Characteristics of the buffalo IgH CDR3 repertoire

**Figure 4:**
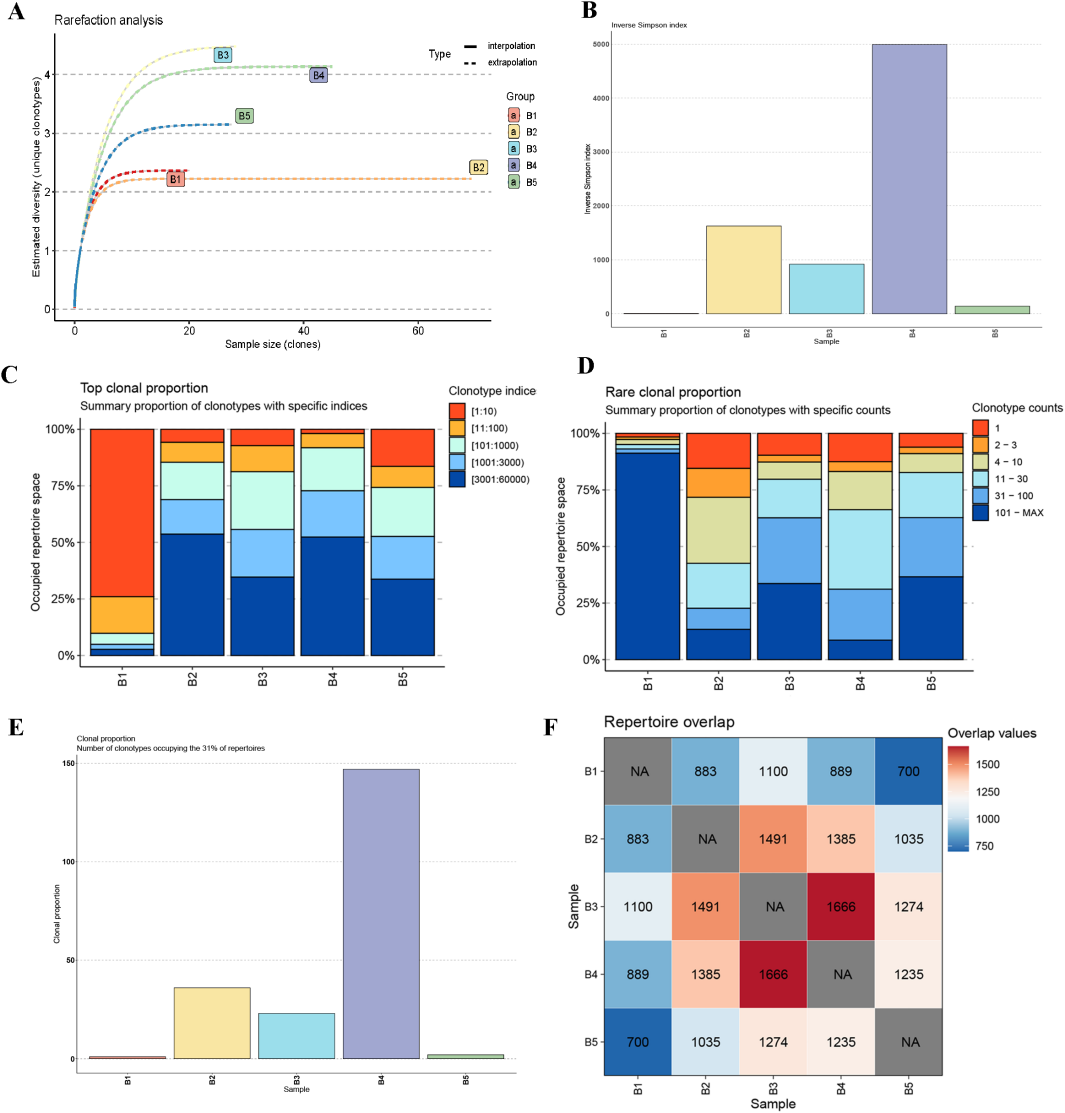
Diversity, clonal distribution, and repertoire overlap analysis of IgH CDR3 sequences in five buffalo spleen samples. **(A)** Rarefaction analysis of IgH CDR3 repertoires; **(B)** The inverse Simpson index analysis; **(C)** Top clonal proportion analysis; **(D)** Rare clonal proportion analysis; **(E)** Clonal proportion analysis; **(F)** Repertoire overlap analysis.

The IgH CDR3 repertoires were obtained from five buffalo spleen samples. Across these samples, the total sequencing reads ranged from approximately 8 to 8.1 million, with successful alignment rates to our custom-built IgH germline reference database between 12.88% and 27.30%. The clonotype count varied from approximately 44,000 to 131,000, while the unique clone count ranged from around 14,000 to 53,000. The proportion of unique clones relative to total clones ranged between 32.3% and 45.9% (**Table 2**). Rarefaction analysis (**Figure 4A**) of the IgH CDR3 repertoires from the five buffalo spleen samples revealed that all samples reached a plateau, indicating sufficient sequencing depth to capture the majority of clonotypes present.

**Table 2:** Summary of sequencing data and clonotype characteristics from buffalo IgH CDR3 repertoires.

Sample	Total sequencing reads	Successfully aligned reads	Clonotype count	Unique clone	Unque/total clone
Buffalo1	8046744	1832517 (22.77%)	44716	14464	32.3%
Buffalo2	8006749	1031164 (12.88%)	98529	43235	43.9%
Buffalo3	8010989	1808047 (22.57%)	131778	53751	40.8%
Buffalo4	8136506	1316264 (16.18%)	107142	47677	44.5%
Buffalo5	8014511	2187835 (27.30%)	90705	41620	45.9%

Subsequent analyses provided further insights into the IgH CDR3 repertoire diversity and clonal distribution across the five buffalo samples. The inverse Simpson index (**Figure 4B**) revealed that Sample B4 exhibited the highest diversity, while Sample B1 had the lowest, with notable differences observed among all samples. The top clonal proportion analysis (**Figure 4C**) showed that the top 10 clones in Sample B1 accounted for a significantly higher proportion of the repertoire compared to the other samples, where the top 10 clonal proportions were more evenly distributed. The rare clonal proportion analysis (**Figure 4D**) corresponded to this, showing that clones with clonotype counts lower than 30 dominated the repertoires in Samples B2 through B5. Clonal proportion analysis (**Figure 4E**), which examined the number of clonotypes occupying 31% of the repertoire, showed a pattern consistent with the inverse Simpson index, with Sample B4 having the highest value and Sample B1 the lowest. Finally, repertoire overlap analysis (**Figure 4F**) demonstrated that shared clones were present between all pairs of samples, with the highest number of shared clones (1666) between Samples B3 and B4, and the lowest (700) between Samples B1 and B5.

**Figure 5:**
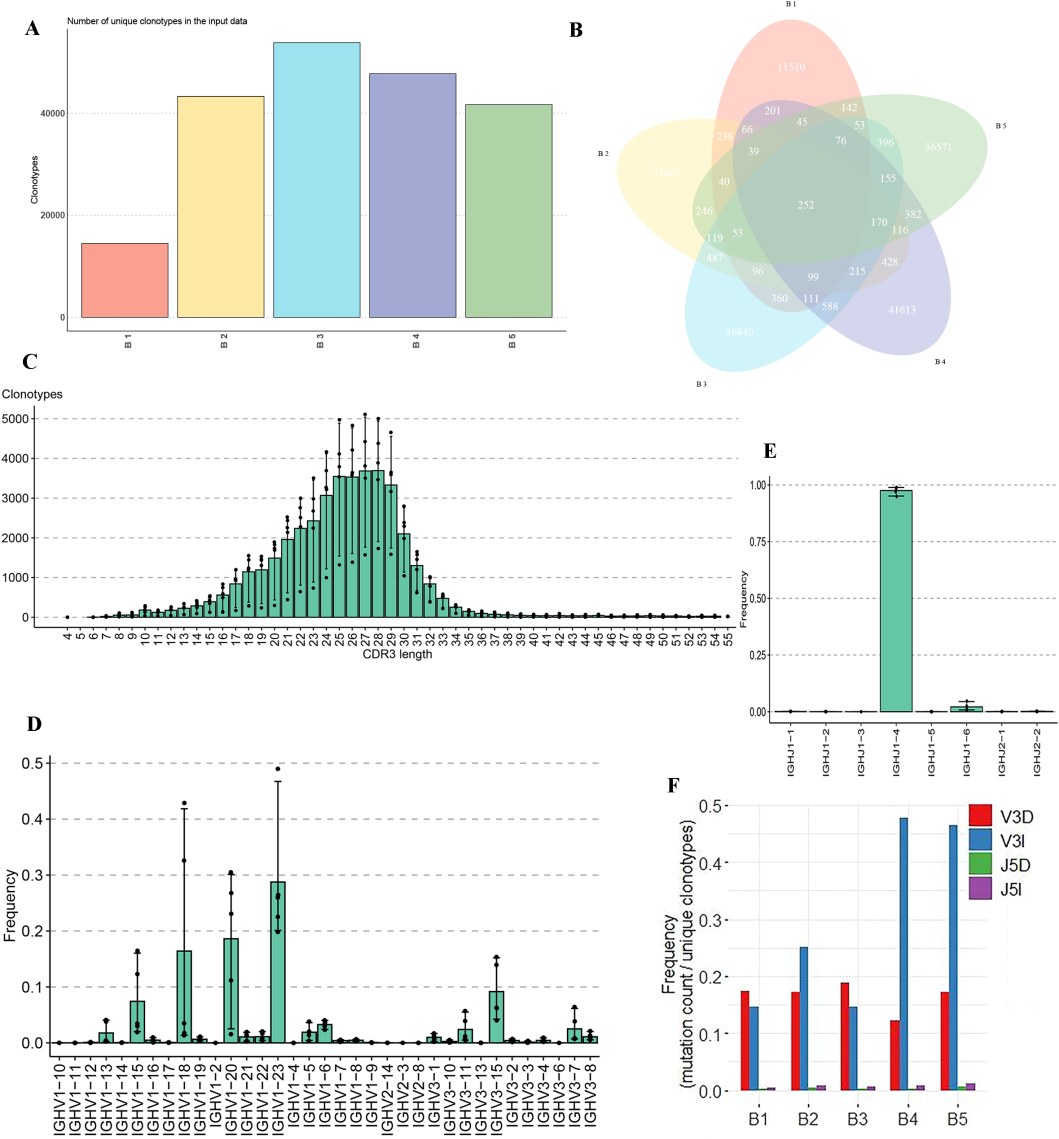
Analysis of unique clonotypes, CDR3 length distribution, V and J gene usage, and junctional diversity in the IgH CDR3 repertoire of buffalo. **(A)** Quantification of unique clonotypes; **(B)** Venn diagram illustrating the 252 shared clonotypes across the five samples; **(C)** Distribution of CDR3 lengths in the buffalo IgH CDR3 repertoire; **(D)** Usage frequency of V genes in the IgH CDR3 repertoire, **(E)** Usage frequency of J genes in the IgH CDR3 repertoire; **(F)** Analysis of amino acid insertions and deletions.

Further analysis focused on the unique clonotypes identified in each sample. As shown in **Figure 5A**, the unique clonotypes for each buffalo sample were identified and quantified. A total of 252 shared clonotypes were found across the five samples (**Figure 5B**). The distribution of CDR3 lengths in the buffalo IgH repertoire exhibited a bell-shaped curve, with a predominant length of 25–28 amino acids (**Figure 5C**). Moreover, ultra-long sequences were identified in the IgH CDR3 region of buffalo, with an average of 168 unique clones per sample having a CDR3 length greater than 50 amino acids, with the longest reaching 70 amino acids (**Supplementary Table 4**). Based on annotation results, we identified 54 V genes and 8 J genes on the buffalo IgH locus, and from our sequencing data, 33 V and 8 J genes were detected (**Supplementary Table 5**, **Supplementary Figure 5**). Notably, IgHV1-23, IgHV1-20, and IgHV1-18 were the most frequently used V genes, while IgHJ1-4 was the most frequently used J gene (**Figures 5D, E**, **Supplementary Figure 5**). Additionally, analysis of amino acid insertion and deletion revealed a high frequency of insertions and deletions at the 3' end of the V gene, suggesting significant junctional diversity in this region (**Figure 5F**).

## Discussion

Understanding the diversity of the Ig immune repertoire is crucial as it directly influences the ability of an organism to mount effective immune responses against a wide array of pathogens, thereby playing a pivotal role in the adaptability and survival of the species (26, 27). The number of V(D)J genes is positively correlated with the diversity of the Ig immune repertoire (28). In this study, we identified a total of 54 VH genes within the buffalo immunoglobulin heavy chain locus, of which 19 are functional. Comparatively, in cattle, the IgH locus consists of 48 IgHV genes, with only 11 being functional (29). Meanwhile, the yak exhibits 42 VH genes, of which only eight are functionally active (13). These differences indicate that buffalo may possess an enhanced potential for antibody diversity, particularly in the context of V gene rearrangement, when compared to both cattle and yaks.

Nearly all mammals (with a few exceptions) possess genes encoding IgM, IgD, IgG, IgE, and IgA (13, 30–32). Buffalo, as members of this consensus group, have also been found to possess these five immunoglobulins. Additionally, multiple IgG subtypes have been detected in buffalo, which is similar to most other mammals (**Figure 1A**). The gene maps for the buffalo heavy and kappa chain loci exhibit the translocon pattern commonly observed in vertebrates, with the 5′ region encoding variable segments and the 3′ region containing constant region elements (33). However, the transcription direction of the λ chain genes differs from the expected orientation, with certain λ chain genes showing reversed transcriptional orientation compared to the typical arrangement. Notably, we identified a segment in the V region of the buffalo containing several V genes with reversed transcriptional orientation. Similarly, in yak, the downstream V genes have an opposite transcriptional direction relative to the chromosome (13). It remains unclear whether these reversed V genes are the result of evolutionary selection, random events, or possibly errors in chromosomal assembly. If these are not due to technical artifacts, further investigation into how these reversed V genes contribute to immunoglobulin rearrangement could provide valuable insights. For instance, studies on the mammalian TRB locus indicate that reverse V genes, such as the conserved TRBV30 gene, have been preserved across species, suggesting a regulatory role in adaptive immunity. Their unique rearrangement patterns and preferential utilization, possibly due to their proximity to the D gene, highlight their functional significance compared to forward V genes (34).

The Igλ genes in many species followed the (Jλ-Cλ)n pattern. The genomic organization of Igλ was structured as Vλn-(Jλ-Cλ)_7_-Vλn in horses, Vλn-(Jλ-Cλ)_6_ in cattle, Vλn-(Jλ-Cλ)_3_-Jλ_4_ in pigs, Vλn-(Jλ-Cλ)_2_ in sheep, and Vλn-(Jλ-Cλ)_2_ in goats (12, 14, 15, 29, 35). In line with these species, buffalo also exhibit a similar Igλ gene structure, possessing three (Jλ-Cλ) units. Like cattle, sheep, and goats, buffalo retain this (Jλ-Cλ) structure, which may reflect a conserved evolutionary mechanism in these ruminants, contributing to the maintenance of antibody diversity and immune response. It is noteworthy that yaks, despite being ruminants, possess a more expanded genomic structure compared to other species, described as Vλn-(Jλ-Cλ)_2_-(Jλ)_2_-(Jλ-Cλ)_5_-Vλ_2_-Cλ-Vλ (13). This difference suggests that even among closely related species, significant evolutionary changes can occur in the genomic organization of Igλ genes. It may further imply that the Igλ genes of different ruminants have followed distinct evolutionary pathways to adapt to specific environmental and pathogen pressures.

The buffalo genome contains 71 Vλ genes, a number significantly higher than that in humans and mice. In contrast, other herbivores species, possess up to 53, 90 and 125 germline Vλ genes in camel, sheep and cattle (29, 36, 37). However, only a small subset of Vλ genes contributes to the majority of rearrangement events (38, 39). This observation suggests that the diversity of the λ chain may primarily arise from mechanisms such as somatic hypermutation and gene conversion. In sheep, somatic hypermutation has been shown to introduce more than 1.1 nucleotide changes per 1,000 bases in certain Vλ regions (39), indicating its substantial role in diversifying the immunoglobulin repertoire. Additionally, the presence of numerous pseudogenes supports the hypothesis that gene conversion contributes to the diversification of Ig gene repertoires (40, 41). This process has been well-documented in other species, such as rabbits and mice, where sequence replacements from homologous pseudogenes introduce variability in many V genes (42, 43). Although we did not investigate the rearrangement of the buffalo λ chain in this study, the high number of Vλ genes, along with the presence of pseudogenes, suggests that similar mechanisms may contribute to the diversity of the buffalo Igλ repertoire.

The hypervariable region of IgH CDR3 is crucial for B cell binding to antigen epitopes, with the length and amino acid composition of CDR3 in immunoglobulin chains influencing the breadth and depth of the adaptive immune response (44, 45). Current knowledge indicates that the most common IgH CDR3 lengths are predominantly 11-13 AA in mice (46, 47), 13-15 AA in bats (23), 16-17 amino acids (AA) in humans (30), 19 AA in cattle (11), and 20-23 AA in yaks (13). Remarkably, our study identifies buffalo IgH CDR3 lengths predominantly ranging from 25-28 AA. This extended CDR3 length may enhance the diversity of antigen binding sites, potentially allowing buffalo to mount a more robust immune response to a wider array of pathogens. Additionally, we have observed a trend that the length of the Ig heavy chain CDR3 appears to correlate with the body size of the species. However, this relationship may not be consistent across all species, as there are some exceptions.

Buffalo possess ultra-long CDR3H, with the longest sequence reaching 70 amino acids, which is longer than those previously reported in yaks (13), sheep (36) and cattle (11). Previous studies have shown that the length of CDR3H in cattle can reach up to 65 amino acids, while the average CDR3H length in yaks was reported as 58.0 ± 15.3 bp, indicating that the CDR3H region of buffalo Ig may exhibit greater variability. The ultra-long CDR3H can form microfold structures, allowing bovine Ig to bind antigens that are otherwise inaccessible to other vertebrates (48). The evolution of ultra-long CDR3H may serve as an additional diversification mechanism for bovines and yaks in response to limited functional VDJ combinations. Alternatively, ultra-long CDR3H may have evolved to optimize antigen binding to rumen microbes or certain bovine-specific pathogens, which are rarely encountered by other vertebrates (48–50). Interestingly, while the “YxYx” feature in the ultra-long CDR3H sequences of cattle and yaks has been observed (11, 13), this feature is less common in the ultra-long CDR3H sequences identified in buffalo in this study. This suggests that different bovine species may exhibit certain structural and functional diversity in their ultra-long CDR3H.

The biased utilization of V/J genes may be directly linked to the individual's specific immune status. For instance, in patients with ankylosing spondylitis, the synovial membrane exhibits a biased repertoire of rearranged heavy chain variable segment genes, with a notable prevalence of VH3 genes (51). Similarly, in COVID-19 patients, observed a pronounced skewing toward specific V gene segments, particularly IgHV4-4 in conjunction with IgHJ6, within clonal B cell receptors (52). In this study, the frequently utilized genes in buffalo included IgHV1-23, IgHV1-20, and IgHV1-18, while IgHJ1-4 was the most frequently used J gene. These data providing a valuable reference background for V/J gene usage in this species. Interestingly, the high frequency of IgHJ1-4 utilization in buffalo bears similarities to the high usage of IgHJ4 genes in humans (with IgHJ4 used at a frequency greater than 40%) (53), which may indicate an evolutionary conservation of certain J gene segments that confer advantageous immune responses across species.

In summary, This study provides a comprehensive analysis of the genomic organization of the IgH, Igκ, and Igλ loci in buffalo, along with a detailed characterization of the IgH CDR3 repertoire. These findings provide a valuable foundation for further understanding the immune responses of buffalo in complex environments and expand our knowledge of Ig repertoire diversity in ruminants.

## Data Availability

The sequencing data have been deposited in the NCBI SRA database under the accession number PRJNA1170869.
