# Supplementary material for: Annotation and characterization of immunoglobulin loci and CDR3 polymorphism in water buffalo (Bubalus bubalis)
Source: Front Immunol. 2025 Jan 20;15:1503788. doi: 10.3389/fimmu.2024.1503788 (PMC11788136; doi:10.3389/fimmu.2024.1503788)
Supplement: Supplementary file 1 [file DataSheet1.docx]

Supplementary Table 1. The upstream primers of the buffalo IGH CDR3 repertoire

| **Name of primers** | **IGHV gene sequences(5'-3')** |
| --- | --- |
| IGHV1 | TGAAATCCCNGCTCAGCATC |
| IGHV1(1-1) | AGCACATACTACCACCCAGC |
| IGHV1(1-21) | AATCCCGGCTCAGTATCACC |
| IGHV2 | NTCCATCAAGAGCCACACCT |
| IGHV2(2-1) | TCCAACAAGAGCCACACCTC |
| IGHV2(2-3、2-8) | CACGCCTCCATCTCAGAGAC |
| IGHV3 | TGGTGGAGCAAAGTAGGCAC |
| IGHV3(1、6、9、14) | AAGTTCNAGGGCAGAGTCAC |
| IGHV3(3-2) | TCCAGGGCAGAGTCATGTTG |

Supplementary Table 2. The downstream primers of the water buffalo IGH CDR3 repertoire

| **Name of primers** | **IGHJ gene sequences(5'-3')** |
| --- | --- |
| IGHJ1-1 | ACCTGAGGAAATGGTGCCCA |
| IGHJ1-2 | AAGGACACGGTGACCGGGGT |
| IGHJ1-3 | AGACGGTGACCGGGGTGCGC |
| IGHJ1-4 | AGACGGTGACCAGGATTCCT |
| IGHJ1-5 | AGCAGACAGCGCCCAGGCTT |
| IGHJ1-6 | AGACGGCGACCCTGAGCCCT |
| IGHJ2-1 | AGACAGTGCCCAGGCTTCCC |
| IGHJ2-2 | GGTGATCCGGATCCTTTGGC |

Supplementary Table 3. Functional discrimination of the IG V genes in water buffalo

| IG V gene | Functionality | Stop codon | Defective RS |
| --- | --- | --- | --- |
| IGHV1-1 | P | ● |  |
| IGHV1-2 | P | ● |  |
| IGHV1-3 | P | ● |  |
| IGHV1-4 | F |  |  |
| IGHV1-5 | F |  |  |
| IGHV1-6 | F |  |  |
| IGHV1-7 | F |  |  |
| IGHV1-8 | F |  |  |
| IGHV1-9 | F |  |  |
| IGHV1-10 | P | ● |  |
| IGHV1-11 | F |  |  |
| IGHV1-12 | F |  |  |
| IGHV1-13 | F |  |  |
| IGHV1-14 | F |  |  |
| IGHV1-15 | F |  |  |
| IGHV1-16 | P | ● |  |
| IGHV1-17 | F |  |  |
| IGHV1-18 | F |  |  |
| IGHV1-19 | P | ● |  |
| IGHV1-20 | F |  |  |
| IGHV1-21 | F |  |  |
| IGHV1-22 | F |  |  |
| IGHV1-23 | F |  |  |
| IGHV2-1 | F |  |  |
| IGHV2-2 | P | ● |  |
| IGHV2-3 | P | ● |  |
| IGHV2-4 | P | ● |  |
| IGHV2-5 | P | ● |  |
| IGHV2-6 | P | ● |  |
| IGHV2-7 | P | ● |  |
| IGHV2-8 | P | ● |  |
| IGHV2-9 | P | ● |  |
| IGHV2-10 | P | ● |  |
| IGHV2-11 | F |  |  |
| IGHV2-12 | P | ● |  |
| IGHV2-13 | P | ● |  |
| IGHV2-14 | P | ● |  |
| IGHV2-15 | P | ● |  |
| IGHV2-16 | P | ● |  |
| IGHV3-1 | P | ● |  |
| IGHV3-2 | P | ● |  |
| IGHV3-3 | P | ● |  |
| IGHV3-4 | P | ● |  |
| IGHV3-5 | P | ● |  |
| IGHV3-6 | P | ● |  |
| IGHV3-7 | P | ● |  |
| IGHV3-8 | P | ● |  |
| IGHV3-9 | P | ● |  |
| IGHV3-10 | P | ● |  |
| IGHV3-11 | P | ● |  |
| IGHV3-12 | P | ● |  |
| IGHV3-13 | P | ● |  |
| IGHV3-14 | P | ● |  |
| IGHV3-15 | P | ● |  |
| IGKV1-1 | F |  |  |
| IGKV1-2 | P | ● |  |
| IGKV1-3 | P | ● |  |
| IGKV1-4 | F |  |  |
| IGKV1-5 | F |  |  |
| IGKV2-1* | P | ● |  |
| IGKV2-2 | F |  |  |
| IGKV2-3* | P | ● |  |
| IGKV2-4 | F |  |  |
| IGKV2-5 | F |  |  |
| IGKV2-6 | F |  |  |
| IGKV2-7 | F |  |  |
| IGKV2-8* | P | ● |  |
| IGKV2-9 | F |  |  |
| IGKV2-10 | F |  |  |
| IGKV2-11 | F |  |  |
| IGKV3-1 | P | ● |  |
| IGKV8-1 | F |  |  |
| IGKV(II)-1* | P | ● | ● |
| IGKV(II)-2* | P | ● | ● |
| IGKV(II)-3* | P | ● | ● |
| IGKV(II)-4* | P | ● | ● |
| IGKV(II)-5* | P | ● | ● |
| IGKV(II)-6 | P |  | ● |
| IGLV1-1 | P | ● |  |
| IGLV1-2 | P | ● |  |
| IGLV1-3* | P |  |  |
| IGLV1-4 | F |  |  |
| IGLV1-5* | P |  |  |
| IGLV1-6 | F |  |  |
| IGLV1-7* | P | ● |  |
| IGLV1-8 | F |  |  |
| IGLV1-9 | P | ● |  |
| IGLV1-10 | P | ● |  |
| IGLV1-11 | F |  |  |
| IGLV1-12 | F |  |  |
| IGLV1-13 | F |  |  |
| IGLV1-14* | P | ● |  |
| IGLV1-15 | F |  |  |
| IGLV1-16 | P | ● |  |
| IGLV1-17 | P | ● |  |
| IGLV1-18 | F |  |  |
| IGLV1-19 | P | ● |  |
| IGLV1-20 | F |  |  |
| IGLV1-21 | F |  |  |
| IGLV1-22 | F |  |  |
| IGLV1-23 | P | ● |  |
| IGLV1-24 | F |  |  |
| IGLV1-25 | P | ● |  |
| IGLV1-26 | F |  |  |
| IGLV1-27 | F |  |  |
| IGLV2-1 | F |  |  |
| IGLV2-2 | P | ● |  |
| IGLV2-3 | F |  |  |
| IGLV2-4 | F |  |  |
| IGLV3-1 | F |  |  |
| IGLV3-2 | F |  |  |
| IGLV3-3 | F |  |  |
| IGLV3-4 | F |  |  |
| IGLV3-5 | F |  |  |
| IGLV5-1 | F |  |  |
| IGLV5-2 | ORF |  | ● |
| IGLV5-3 | ORF |  | ● |
| IGLV5-4 | ORF |  | ● |
| IGLV5-5 | ORF |  | ● |
| IGLV5-6 | P | ● |  |
| IGLV5-7 | P | ● |  |
| IGLV5-8 | F |  |  |
| IGLV5-9 | P | ● |  |
| IGLV8-1 | F |  |  |
| IGLV8-2 | P | ● |  |
| IGLV8-3 | F |  |  |
| IGLV8-4* | P | ● |  |
| IGLV8-5 | F |  |  |
| IGLV8-6* | P |  |  |
| IGLV8-7* | P |  |  |
| IGLV8-8 | F |  |  |
| IGLV8-9 | P | ● |  |
| IGLV8-10 | F |  |  |
| IGLV8-11 | P | ● |  |
| IGLV8-12 | F |  |  |
| IGLV8-13* | P | ● |  |
| IGLV13-1 | F |  |  |
| IGLV13-2 | ORF |  | ● |
| IGLV13-3 | P | ● | ● |
| IGLV(I)-1* | P | ● |  |
| IGLV(I)-2* | P | ● |  |
| IGLV(I)-3 | P | ● |  |
| IGLV(IV)-1* | P | ● |  |
| IGLV(IV)-2* | P | ● |  |
| IGLV(IV)-3* | P |  |  |
| IGLV(IV)-4 | P | ● |  |
| IGLV(IV)-5* | P | ● |  |
| IGLV(IV)-6* | P | ● |  |
| IGLV(IV)-7* | P | ● |  |

“*”: no L-PART1

Supplementary Table 4. CDR3length

|  | **B1** | **B2** | **B3** | **B4** | **B5** |
| --- | --- | --- | --- | --- | --- |
| range | 7-55 | 6-58 | 6-70 | 6-67 | 6-70 |
| average | 25.94±4.46 | 25.01±5.34 | 24.99±5.03 | 25.34±5.62 | 25.07±5.79 |
| >50 AA | 7 | 152 | 75 | 221 | 231 |

Supplementary Table 5.The number of V and J genes detected in the sequencing data

|  | **B1** | **B2** | **B3** | **B4** | **B5** |
| --- | --- | --- | --- | --- | --- |
| V | 29 | 29 | 31 | 30 | 33 |
| J | 6 | 7 | 7 | 7 | 8 |

Supplementary Figure


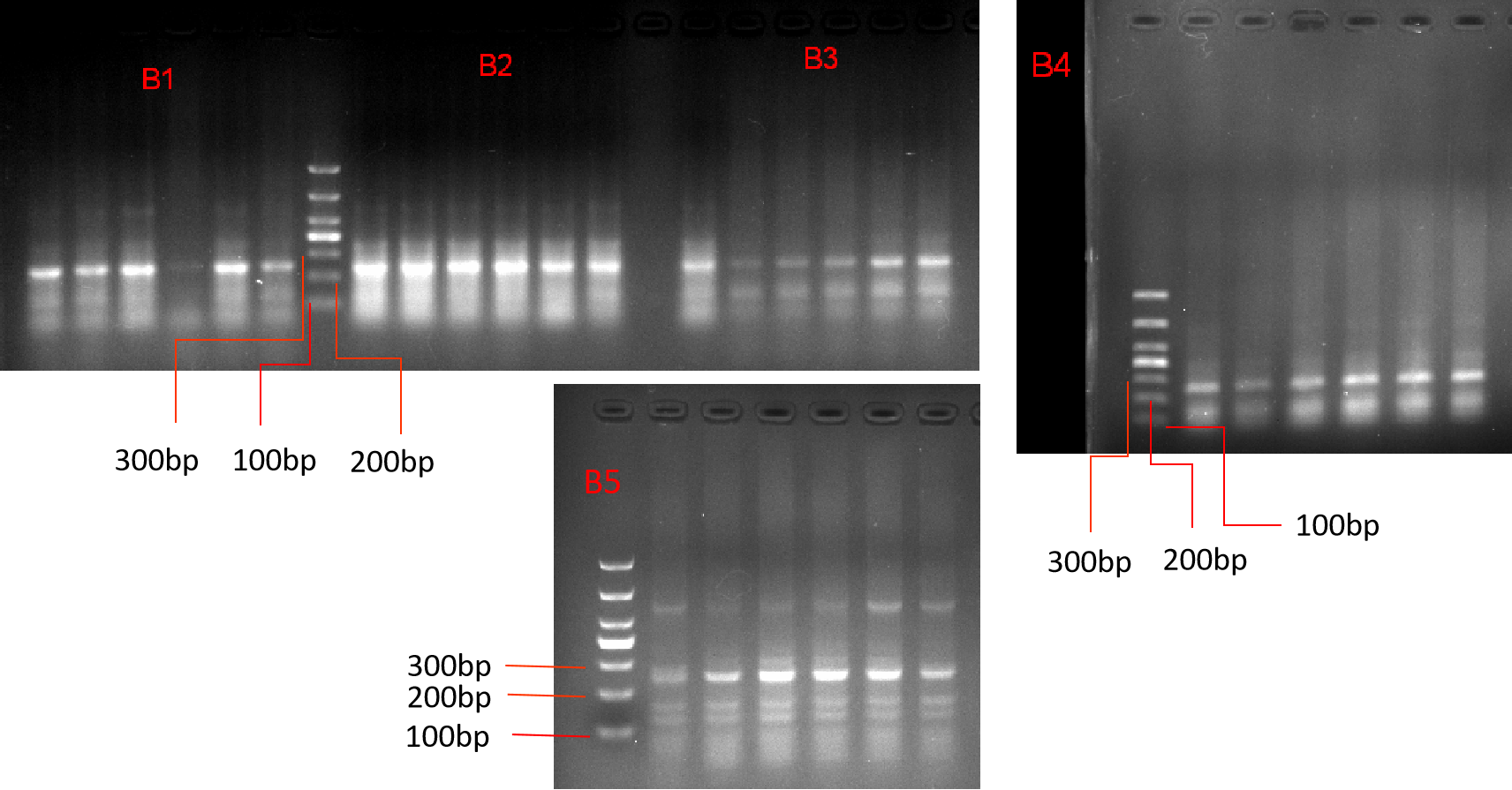


Supplementary Figure1: PCR products of IGH CDR3 receptor library identified by agarose gel electrophoresis.


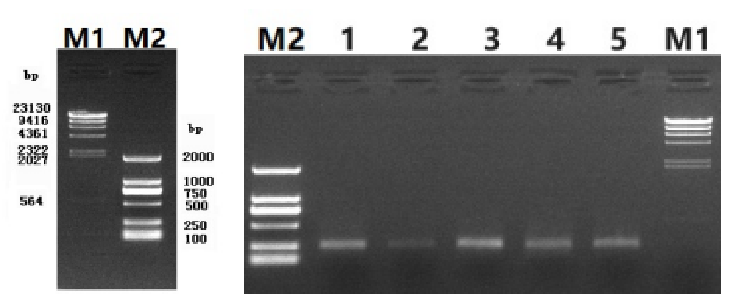


Supplementary Figure2：Identification of agarose gel electrophoresis of the gel recovery product.


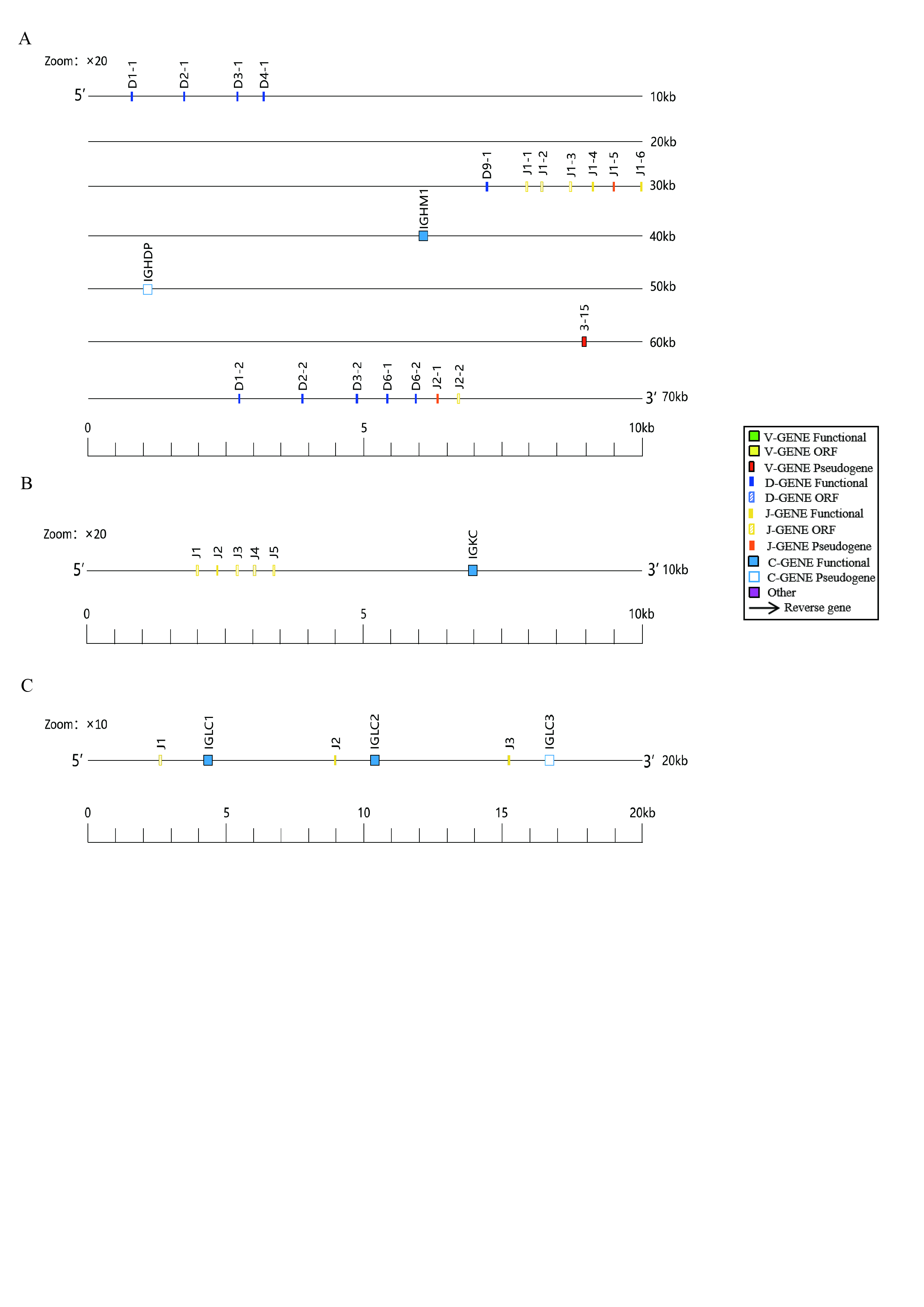


Supplementary Figure3 A:The D-J-C cluster of the water buffalo IGH locus. B:The J-C cluster of the water buffalo IGK locus. C:The J-C cluster of the water buffalo IGL locus.
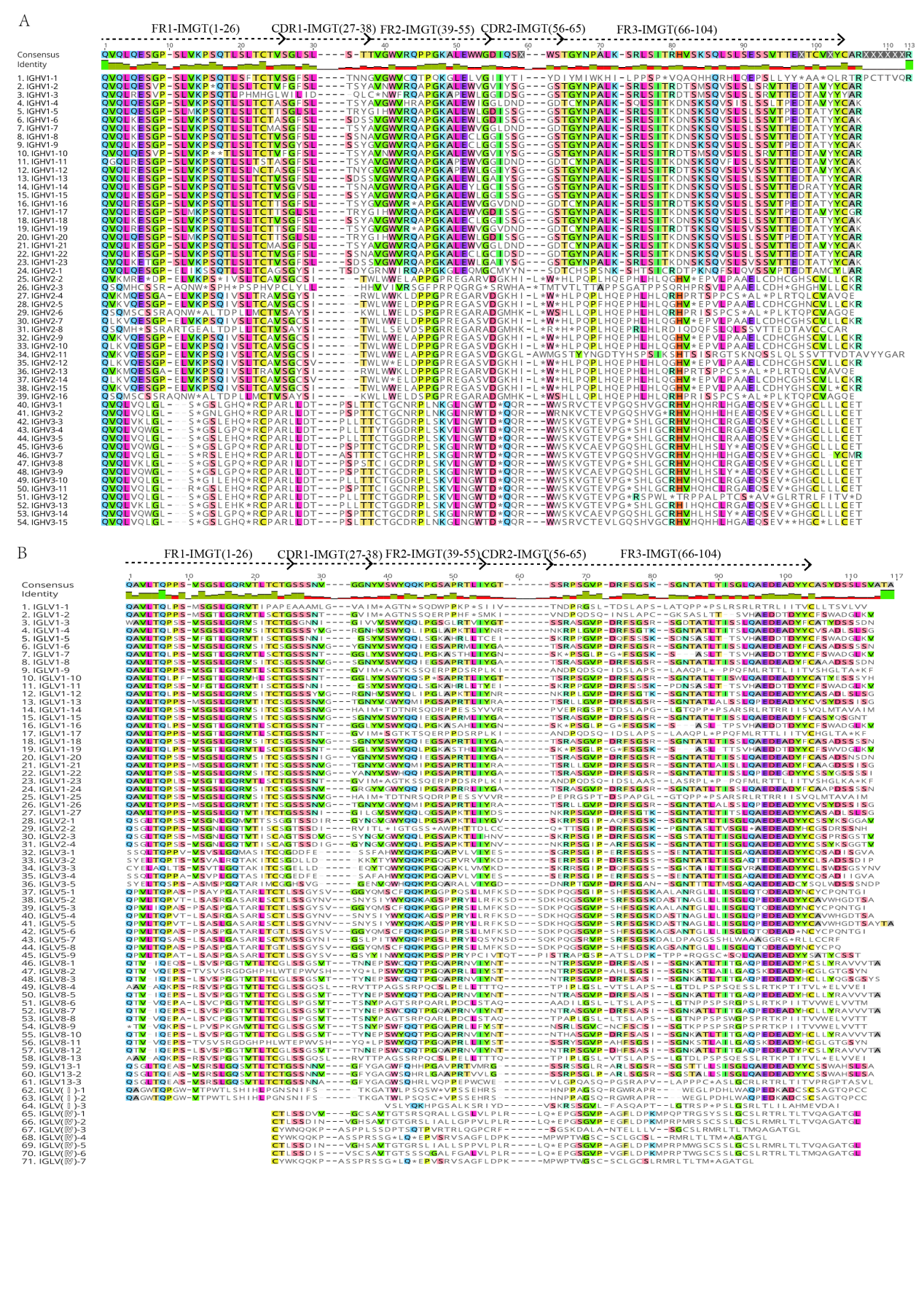
Supplementary Figure4 A:Characteristics and Structural Analysis of the IGHV Amino Acid Sequence Composition in Water Buffaloes. B:Characteristics and Structural Analysis of the IGLV Amino Acid Sequence Composition in Water Buffaloes.


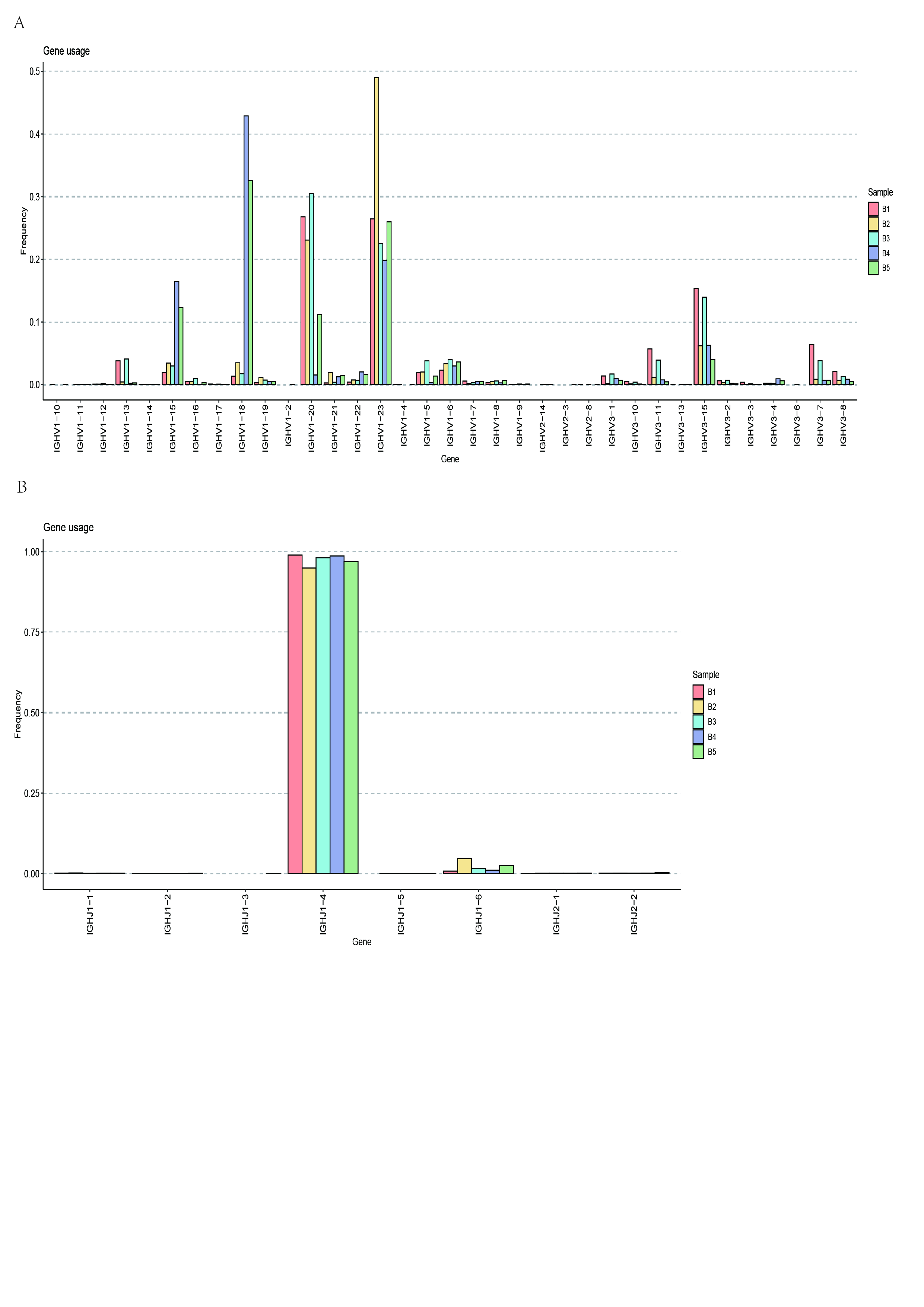


Supplementary Figure5：A: the usage of Buffalo V genes. B: usage of Buffalo J genes.
